# Delivering high-quality biometry in low-resource settings: a practical guide

**Published:** 2025-09-19

**Authors:** Adeyemi Adeola, Andrew Blaikie

**Affiliations:** 1 Consultant Ophthalmologist: Ancilla Catholic Hospital Foundation Eye and Laser Centre, Lagos, Nigeria and International Clinical Fellow: Queen Margaret Hospital, NHS Fife, UK.; 2 Senior Lecturer, University of St Andrews, Scotland, UK.

**Accurate biometry is possible in any setting when a small, well-trained team follows a standardised protocol, audits its results, and keeps its equipment calibrated and well maintained.**

**Figure:**
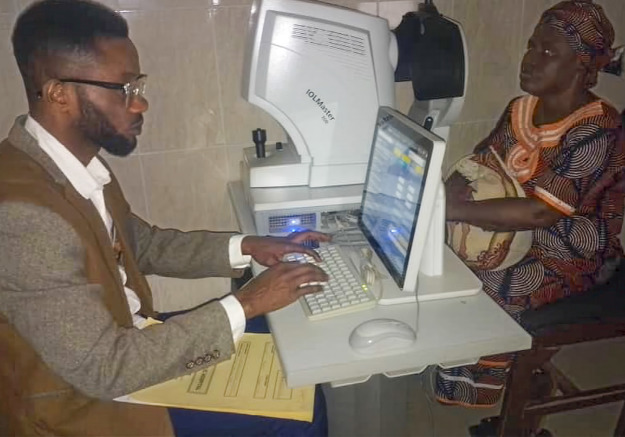
An optical biometer used in a base hospital. NIGERIA

© ANCILLA CATHOLIC EYE FOUNDATION CC BY-NC-SA 4.0

Biometry is the cornerstone of modern cataract surgery because it helps surgeons to select the correct intraocular lens (IOL) power for their needs. Modern biometry measures variables such as axial length, anterior chamber depth, lens thickness, white-to-white corneal diameter, corneal power (keratometry, or K), and effective lens position. A study on parameters influencing refractive outcomes in a high-income setting (the Netherlands) showed that effective lens position contributed 27% to refractive predictive error, axial length 17%, and K measurements 10%.^1^ This may be due to inaccuracies and limitations in measurement and calculation techniques.

In low-resource environments, the risk of such errors rises because staff members often have higher patient numbers and have to work with older machines in a more challenging environment, leading to greater stress. This practical guide explains, step by step, how eye care teams can deliver accurate, repeatable biometry despite these constraints. Accurate biometry supports the wider goal of effective cataract surgical coverage, by optimising postoperative vision for as many patients as possible.

## Choosing practical equipment

### Axial length

When funds are scarce, the first decision concerns axial length measurement. A handheld A-scan ultrasound unit that costs between USD 1,000–3,000 is widely available and robust enough for field work. Adding an inexpensive immersion shell (approximately USD 200) immediately improves accuracy by avoiding corneal compression.

**Figure:**
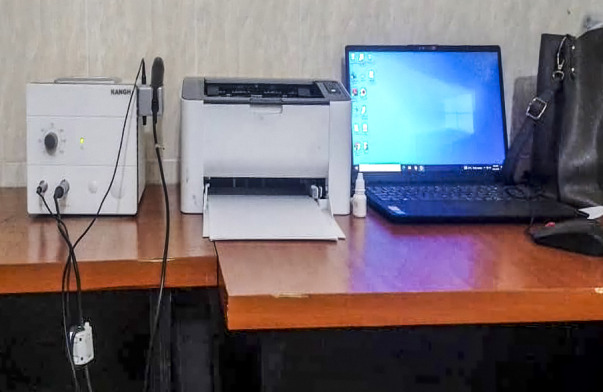
Portable/mobile modular equipment: A-scan, B-scan, pachymetry, ultrasound biomicroscopy. NIGERIA

© ANCILLA CATHOLIC EYE FOUNDATION CC BY-NC-SA 4.0

Optical biometers, such as the IOLMaster, Lenstar, and Eyestar, deliver good precision and speed, but their prices can exceed USD 16,000. One shared optical unit, transported with the surgical team, often represents the best compromise for a cluster of district hospitals. Since there is often limited clinical space in such settings, it is necessary to prioritise portability of the equipment, in addition to the safety of the equipment and the operating environment.

### Keratometry

A base hospital that can rely on mains electricity may invest in a tabletop autokeratometer, or autorefractor keratometer, for faster keratometry, while outreach teams can use manual keratometers (approximately USD 800) or even some newer smartphone attachments. Some may choose to transport an autokeratometer as was the case with the team in Uganda (see article in this issue).

## Human resources: small team, big impact

**Equipment alone does not guarantee accuracy.** A dedicated biometry team made up of two to four motivated technicians can produce results that match those of large advanced centres.

**Clear role separation** allows surgeons to focus on the operating theatre while maintaining confidence that every IOL calculation is sound. For example, one team member positions the patient, captures the measurements, and checks quality, while a second member records the data, keeps batteries charged, and disinfects probes so that the flow of patients never slows down.

**Train team members to adequately perform various aspects of biometry.** This will prevent interruptions to biometry services when a team member is unwell or on leave. Use checklists and protocol sheets (see panel) to ensure each team member records information appropriately.

**Provide regular refresher training for team members** so that they are up to date with of advancements in biometry protocols.

**Figure:**
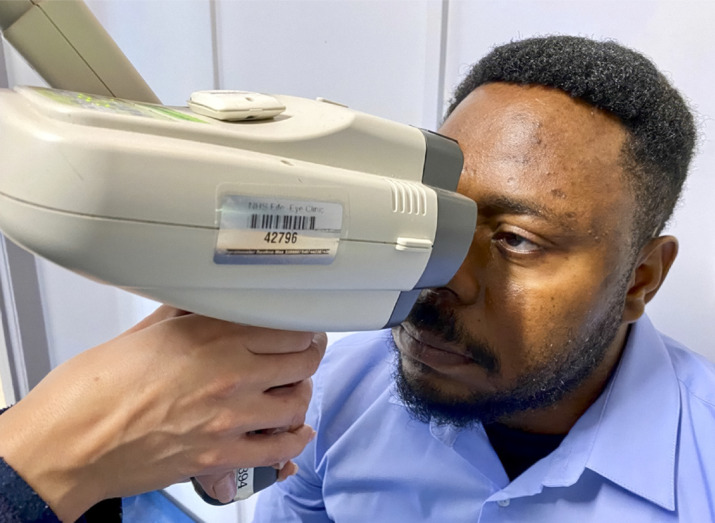
Using a portable keratometer. NIGERIA

© ANDREW BLAIKIE – VHK CC BY-NC-SA 4.0

**Figure:**
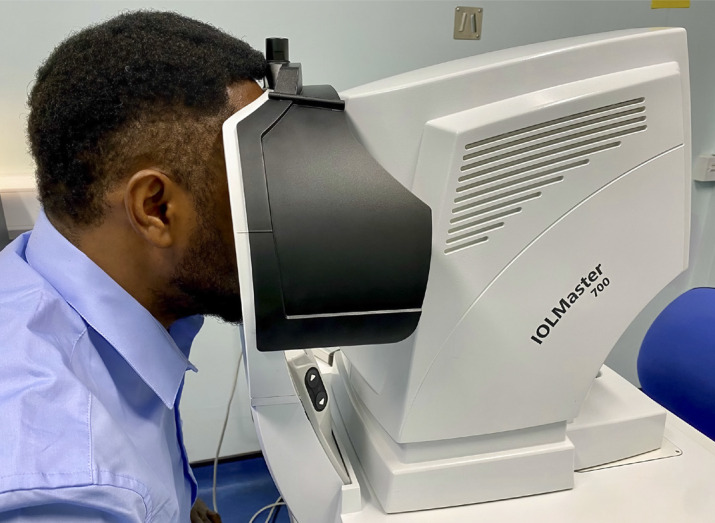
Using an optical biometer. NIGERIA

© ANDREW BLAIKIE – VHK CC BY-NC-SA 4.0

## Standard workflow at the base hospital

Consistency begins with the operating environment and set-up. A small darkened cubicle fitted with a chair with a firm headrest stabilises the patient's gaze and eliminates glare.

Ideally, a technician would calibrate the A-scan with a test block and perform a zero check on the keratometer. Immersion ultrasound is preferred because it removes the risk of pressing the probe against the cornea, but contact ultrasound is acceptable provided at least five traces are recorded for each eye and the standard deviation is kept below 0.1 mm.

Contact ultrasound should ideally not be performed on the day of the operation. If this cannot be avoided, then try to ensure that it is performed more than four hours before the operation, as disruption to the corneal epithelium can impair the view during surgery.

Keratometry should be taken three times and the average used for the calculation. Enter results immediately into the electronic record or a bound logbook to avoid transcription errors. Any axial length difference greater than 0.3 mm, or any unexplained asymmetry in corneal power, should trigger a repeat measurement or a review by a senior team member. By adhering to this sequence, the team soon finds that accuracy does not slow them down; it simply becomes the routine.

## Outreach workflow: portability and reliability

Cataract outreach brings care to people who might never reach a hospital, yet it exposes the team and equipment to dust, heat, and erratic electricity supply. Essential kit should include a battery or electric-powered A -scan (whichever is available and suitable), a manual or handheld autokeratometer, a small tablet or printer (optional), spare batteries, and alcohol wipes. When water baths for immersion are impractical, the team can rely on contact ultrasound with plans for a slightly myopic offset of 0.25 dioptre (D) to compensate for possible corneal compression. With practice, two operators can measure twenty patients an hour without losing quality. At the end of each session, clean the probes, back up the data to an encrypted memory stick, and charge every battery from a solar power bank, ready for the next village or outreach visit.

## Ensuring repeatability and accuracy

**Keratometry.** The accuracy of keratometry depends on an undisturbed tear film and so it is important that readings are taken before instilling anaesthetic eye drops or touching the cornea with a tonometer; patients must also not wear contact lenses for 2 weeks or more beforehand. This prevents changes in the tear film that will affect the readings. In addition, correct focusing and proper centring of the mires are essential. Three measurements are taken and averaged, but the eyes are rechecked if the corneal powers differ by more than 1.5 D or if either eye falls outside the 40.0 D to 48.0 D range. When scars distort the mires, the team may use the fellow eye (second eye prediction refinement) or, where available, a topographer, always remembering that accuracy in one parameter cannot compensate for inaccuracy in the other.

**Axial length.** The key to reliable examinations is alignment of the probe with the eye being measured. The probe or optical beam must point directly at the fovea, otherwise the reading will be falsely long or short. The patient should fixate on the probe light with the eye being examined. If the patient finds this difficult, it is helpful to have them fix on a distant target with the other eye. The operator should select the lowest gain that still shows clear echoes from the cornea, anterior lens, posterior lens, and retina. A missing scleral spike indicates that the beam has wandered into the optic nerve and the trace must be discarded. Only the five most consistent traces are averaged, and the operator repeats both eyes if the results differ by more than 0.3 mm or if either eye measures shorter than 22 mm or longer than 25 mm. Immersion is strongly advised for such extremes, because even a 0.1 mm error can shift the chosen IOL by 0.5 D in a short eye or by 0.25 D in a highly myopic eye.

## IOL power prediction formulae

More modern formulae have replaced the old SRK II conversion charts, and many of these new formulae are available free of charge online. For eyes shorter than 22 mm, most surgeons favour Hoffer Q, Holladay II, or the Barrett Universal II. For eye lengths between 22 mm and 26 mm SRK/T, Hill-RBF, and Barrett remain reliable; Haigis, Barrett True-Axial, and Hill-RBF are considered to perform better for eyes longer than 26 mm. Whichever formula is chosen, the surgical team should audit its outcomes every quarter and adjust the A-constant for their preferred IOL model until the mean absolute error sits comfortably below 0.5 D.

## Data capture and the audit cycle

A prospectively collated spreadsheet can support auditing (see article on auditing in this issue). Discussing these figures with the entire team turns numbers into learning. If the mean error creeps above 1.0D, the technicians retrain; if a consistent bias appears, the A-constant is adjusted accordingly. Publishing local results keeps the focus of the team on maintaining high-quality care, and that builds trust with patients. It may also help persuade administrators that an optical biometer or a second keratometer is money well spent.

## Cost-saving and sustainability tips

High-income hospitals often replace equipment long before it fails, so partnerships with high-income hospitals can yield refurbished ultrasound units and keratometers at a fraction of their original cost. Sharing an optical biometer between district centres, couriered with the surgical packs, spreads both the expenses and the benefits. Solar chargers with batteries can guard against power cuts. The guiding principle is that technology must serve the patient, not the other way round. For consumables, consider bulk purchasing or shared services.

## Maintenance

Poor maintenance due to lack of technical support is an important contributor to biometry services not being sustained. It is important that any devices chosen can be serviced locally, calibrated daily, and backed up by low-tech alternatives when power fails or if the cataract is too dense for optical methods.

## Key take-home messages

Accurate biometry is possible in any setting when a small, well-trained team follows a standardised protocol, audits its results, and keeps its equipment calibrated and well maintained. Contact ultrasound remains reliable provided the operator avoids corneal compression and respects the standard-deviation limits. Immersion or optical methods improve outcomes for very short or very long eyes but are only worthwhile if the data are recorded correctly and reviewed regularly. Low-cost innovation and shared resources can help ensure that all communities can access optimum results from modern cataract surgery.

**Practical tips**

**How to reduce errors while using a keratometer**

1. Calibrate and check the accuracy of the keratometer.
2. Use a dedicated single instrument that is known to be accurate.
3. Don't touch the cornea beforehand, and ensure a good tear film.
4. Adjust the eyepiece to bring the central cross-hairs into focus.
5. Make sure that the patient's other eye is occluded and that the cornea is centred.
6. Take an average of three readings, including the axes.
7. If high or low results are encountered (< 40.0 D or > 48.0 D), it is advisable to have a second person check the measurements.
8. Repeat if the difference in total keratometric power between the eyes exceeds 1.5 D.
9. In a scarred cornea, use the fellow eye or average the results.

**How to reduce errors while using A-scan**

1. Check machine calibration and set for the correct velocity setting (e.g., cataract, aphakia, pseudophakia).
2. The gain should be set at the lowest level at which a good reading is obtained.
3. Apply topical anaesthesia.
4. The four echoes from cornea, anterior lens, posterior lens, and retina should be present and of good amplitude. Misalignment along the optic nerve is recognised by an absent scleral spike.
5. Maintain eye alignment by asking the patient to fixate on the light from the probe to avoid underestimation.
6. Avoid any cornea compression – don't push too hard.
7. Take the average of the five to ten most consistent results giving the lowest standard deviation (SD) – ideally, < 0.06 mm. Reject any axial-length SD > 0.1 mm.
8. Always measure both eyes and repeat if the difference between eyes is greater than 0.3 mm, or if consecutive measurements differ by more than 0.2 mm.
9. Look out for extreme readings and unexpected values – very short (less than 22 mm) or very long (more than 25 mm).
10. There must be a weekly comparison of inter-observer differences.
11. The immersion method increases accuracy in long/short eyes, where a scleral (Prager) shell is used with the patient in a supine position. It is better to opt for immersion or contact-plus-myopia when precision tools are lacking.
